# Lessons for science and technology policy? Probing the Linkedin network of an RDI organisation

**DOI:** 10.1007/s43545-022-00586-3

**Published:** 2022-12-08

**Authors:** Evi Sachini, Konstantinos Sioumalas-⁠ Christodoulou, Nikias Bouras, Nikolaos Karampekios

**Affiliations:** 1 National Documentation Centre, 48 Vas. Konstantinou Str., 11635 Athens, Greece; 2 Department of History and Philosophy of Science, National and Kapodistrian University of Athens, Athens, Greece

**Keywords:** Data science, RDI system, LinkedIn, Social media, Network, Greece

## Abstract

In this paper, we seek to examine the network of the Greek National Documentation Centre (EKT) as formed by its LinkedIn followers. By applying specific data collection and processing techniques, we explore the network of all the individuals that follow EKT’s LinkedIn page. Significant manual and automatic approaches have been implemented with regard to data extraction, data curation and data homogenization. The aim is to identify the network’s advancement over time, the institutions involved and the countries. The timeframe of the study spans from when the relevant LinkedIn page was constructed in 2015 to 2020. Findings indicate that there is a steady increase in the number of new followers, peaking in 2020. On an international scale, the evolution of the network of followers is imprinted and distributed in worldwide maps. In total, 68 countries have followed EKT over the examined time period. Also, in terms of followers’ institutional sector the Business Sector (BES) stands out (46.5%). Higher Education (HES) and Government Sector (GOV) are associated with 26.4 and 22.2% of the followers, respectively. Lastly, this paper provides a first institutional and country-level mapping of who constitutes the organisation’s interlocutors in the national and global RDI ecosystem.

## Introduction

### The rise of social media

During the last few decades, social media (also referred to as Social Networking webSites—SNS (Chang 2017)) have become a key medium for people and organisations to engage in communication inter alia for an increasing number of purposes. Ranging from personal issues and commercial interests to job hiring, political discussion and activism, social media platforms have become privileged spaces for heated discussions. Further, it would not be an exaggeration to say that social media have become an ideal trove for the outing of (mis)information dissemination. As such, social media have attracted not only the interest of potential users but also of analysts alike. With an ever increasing information volume, analysis presents a particular interest for understanding patterns of behaviour and subsequent actions taken by individuals and communities of people, etc., with vested interests.

The easiest ways to pinpoint this genre of websites is to consider that all of them revolve around a network of connections (Leonardi 2013). The timelessness of the importance of social media in policy is a fact that acknowledges their universal reach to guide decisions (Shirky 2011). Their present increasing availability and use appear to add more to this power. This growing use of social media is in the process of changing how news is produced, disseminated and discussed. Nielsen and Schrøder (2014) studied the relative importance of social media for accessing, finding and engaging with news. In respect of this, a set of similarities in terms of the growing importance of social media as part of people’s cross-media news habits was found. Later studies (Utz 2016; Hemsley et al. 2018), sought to examine the overall beneficial character of social media. On the former, users have become more aware of issues in the society they live in. Also, according to Siddiqui et al. (2016) social media does provide people with positive effects such as emotional support, exchange of information/news and/or professional progress in a more time-efficient manner between connections.

### Typology of key social media outlets

The type of people to connect with in each social platform is the best way to categorise them. Some of the most common social media platforms are Facebook, Twitter and LinkedIn. Facebook can be called a tool for many purposes. According to Mazman et al. (2010), most of those purposes can be assumed to be connected with one’s social circle (communication with friends or even making new ones), their work (online sharing of project data) or just daily activities (playing games and/or getting in touch with other similar hobbyists). However, as a platform as widely used as Facebook, it can be used to study its continuously increasing size of data. As stated by Gendronneau et al. (2019), the data that can be found on the platform can serve as a manner of guiding marketer’s ads. Since the platform is capable of serving specific ads to users with particular interests, by definition it must have the knowledge of what these interests are. But knowledge of the interest in a list is only the tip of the iceberg. When it is paid, Facebook can manage to speak about its user’s location. Indeed, such data scraped from what users have submitted in their “general information” of their profiles, and validated by posts and GPS in their devices, can be useful for studies dealing with the measuring of labour mobility, interests (long lists that paying people can use to filter users with), behaviours (sportiness or movie/TV likes and dislikes) and demographics (gender, age, education level, relationship status or workplace) (Gendronneau et al., 2019).

Twitter has increasingly infused itself into daily life. It plays an important role in political events and journalism—many people, including journalists themselves, now learn about breaking news from their Twitter feed. It is also integral to contemporary public relations and the promotion of products and celebrities, as well as in the construction of social networks and communities related to many different interests (Lupton 2014).

A key difference here between social media and social network sites is the design of the former to be explicitly public geared towards interactive multicasting (Murthy 2018). And when combined into one platform—i.e. Twitter—Murthy (2018) claims that the result is a “real-time public, many-to-many broadcasting” that is only limited by the number of one’s followers. Indeed, Twitter is increasingly being used to share near-real-time analysis of emergent and sometimes hazardous events. This is reflected upon studies including geological events (Hicks 2019; Earle 2010; Sakaki, Okazaki, and Matsuo 2010), activist movements (Li et al. 2020; Xiong et al. 2019; Theocharis et al. 2015; Tremayne 2014), patient support groups, health care workers and medical researchers (Curtis et.al, 2018; Pershad et al., 2018; Hawn 2009; McIver et al. 2015). Alternatively, Twitter can be used as a technical support tool (Lam and Hannah 2017), an altmetric indicator (Thelwall et al. 2013) or deployed as a source of material financial information (Dredze et al. 2016).

Moreover, there is a website explicitly for professionals, LinkedIn. LinkedIn is a professional networking platform that also operates via websites and mobile apps. Launched in 2003, the platform includes employers posting jobs and job seekers sharing their education and employment history (Gendronneau et al. 2019). It is more popular with people with higher pay and with more advanced education (Kim et al. 2017), while known as a platform for professional self-promotion and graduate job recruitment. As a social networking website it can be a valuable tool in 21st century career development learning more broadly, used for research into industry opportunities, structures and norms, professional network development and informal learning (Bridgstock 2019).

It is commonplace that social media has changed how to brand yourself, apply for jobs that are professionally satisfying and challenging, and provide career progression. Indeed, number of studies, focussing mainly on LinkedIn, address social media marketing strategies relating to career advancement (McCabe 2017; Case et al. 2012; McCorkle and McCorkle 2012; Von Rosen 2012), while others opt to identify the utility of Linkedin on selection and recruitment (Koch et al. 2018; Subhani et al. 2012; Zide, Elman et al. 2014; Blacksmith and Poeppelman 2014).

On a macroscopic level, LinkedIn moves beyond the passivity of advertising to its users towards actively structuring digital labour markets, in which it strategically includes universities and its constituents. As such, it is gradually building a global marketplace for skills to run in parallel to, or instead of university degrees (Komljenovic 2019). In addition, LinkedIn data are best at representing skilled labour in the knowledge-intensive, and tradable sectors (Zhu, Fritzler, and Orlowski 2018). The World Bank Group–LinkedIn partnership is indicative of the importance of LinkedIn in measuring its impact on crucial sectors of society (education, economy, business, etc.). This partnership is a three-year effort between the WBG and LinkedIn to investigate the extent to which LinkedIn’s data can inform policy (Zhu et al. 2018).

Importantly, data generated by social media platforms can be used to support social research and policy. Analysis of such data can provide indications of information seeking behaviour, indications of public opinion and understanding of the network entailed, be that individuals, organisations, etc. (Klašnja et al. 2018).

### Answering questions and making policy out social media

Academics, analysts and policy officials have been quick to pick up social media as a potential trove of data and content that could be analysed and exploited for a number of relevant questions. Ranging from expanding knowledge about (perceptions on) climate change (Harvard University 2022; Tuitjer and Dirksmeier 2021) to informing policy action (Vydra and Kantorowicz 2021) for health and well-being (Yeung 2018a, b) as well as better entrepreneurship-related choices (Martín-Rojas et al. 2020), such real-time data are increasingly being considered as a central means for answering heated and time-relevant questions. Answering real-time questions on e.g. migration and mobility is yet another example of the data-rich exploitation potential such social media have (Culora et al. 2021).

Research wise, it should be noted that the aspect of social media that makes it more interesting to scientists is the increased efficiency of communication with third parties (i.e. the connections of the specific social media platform), such as the readers of research, or the parties that have submitted a piece of research interesting to a reader (Nat Cell Biol 2018). This extremely efficient manner of communicating with one’s network has even changed the scientific way of work in many cases. Nicholas (2015) explains how scientists using social media sometimes share with their community before asking for peer–peer review of their work. While this approach is not without valid concerns relating to academic integrity, the activation of scientists in areas as accessible as social media is a phenomenon which is making science more popular (Hemsley et al. 2018).

An increasing range of policy fields are exploring data drawn from social media as an informational input for guiding their actions. Banking on accuracy as a means to counter misinformation (Pennycook et al. 2021), examples include the following fields. It can be used as a mechanism for governments to introduce public policies as well as a test-bed for appreciating the impact such policies may have on the electorate (Singh et al. 2020; Driss et al. 2019), to predict future policy issues that are considered to be important on various levels (national, regional, developmental) (Grubmüller 2013) and enhance voting turnout (Poy and Schüller 2020). On health issues, social media data are increasingly being considered as an informational input for health-related policy interventions (Yeung 2018a, b) to measure the public opinion on government reaction to public health issues, referring to COVID-19 (Van Dijck and Alinejad 2020; Yigitcanlar et al. 2020; Liu et al. 2021).

Increased use and uptake of social media is not without downsides, unfortunately. Too large a number of connections points to information overflow leading to the inability to read the posts within their network. In addition, there are enormous security concerns, one of which is privacy considerations (Hemsley et al. 2018; LaPoe 2017). Also, advertisements have become a major user experience nuisance by the sheer number of them (Heches 2014). In fact, social media and traditional sources of advertisement are considered jointly by marketeers (Stephen 2012). Social media are playing a prominent role in the dissemination of (dis- and mis-)information (Venegas-Vera et al. 2020) and conspiracy theories (Erokhin et al. 2022; Theocharis et al. 2021). Countering these points, governmental authorities have also entered the field of social media data analysis. For example, intelligence was among the first to use social media as probes for potential radicalization tell-tales (RAND 2017) as well as inciting violence (Arayankalam and Krishnan 2021). Tax authorities is yet another example (Zhang et al. 2021; Politico 2021).

### Understanding RDI systems through social media

Banking on this increased exploitation of social media data and content for policy purposes, science and technology policy is a field that has also started to make inroads on such data and content for the purposes of understanding patterns, drawing lessons and making recommendations concerning the RDI (Research, Development and Innovation) ecosystem. Indeed, social media platforms have been utilised to explore different aspects of this system. For example, social media have been used to analyse communication processes within online communities (Choi et al. 2012), to identify and establish information exchange between radiation oncology communities (Sarkar et al. 2019), to explore how social media enables innovation activities (De Oliveira et al. 2020) and to examine and study the patterns through which scholarly articles are discussed and shared on social media (Wang et al. 2016). The issue of science communication is herein important for a number of reasons—building support for the uptake of scientific evidence in policymaking, informing people about the science behind the policies and it impacts their life, and improving public understanding of science. Herein, in the case of the COVID-19 pandemic social media were exploited for communicating related scientific and technological literature (Li et al. 2021; Kothari et al. 2021).

Moreover, the field of RDI has been explored in relation to the determinants (and objectives) that induce highly skilled individuals to register on LinkedIn and to develop networks (Baruffaldi et al. 2017), the use of social media to identify and integrate lead users into the process for developing innovative product concepts (Ernst and Brem 2017) and the use of Chinese government microblog accounts as innovations in the public sector (Zheng and Zheng 2014), as well as the overall attention attributed to science issues (Díaz-Faes et al. 2019). Given that data coming from social media are increasing, the number of researchers on RDI that seek to extract useful information from these media will most probably increase in the future.

An aspect that is under-represented in the relevant bibliography concerns the presence of RDI institutional actors in social media. That is, while students of RDI (such as the above) seek to track and collect data for specific aspects of the RDI continuum, very few if any have explored the presence of RDI-related organisations in social media. Given that social media is an entire communication ‘world’ wherein researchers, academics entrepreneurs and policy officials intermingle and exchange information, building awareness and authority, demonstrating authenticity, encouraging engagement as well as providing support to the above communities and learning more about them stand as legitimate reasons for relevant institutions to engage and (more importantly) seek to explore and understand the characteristics of those ‘networked’ with their own digital media. The topic that regard the utilisation of social media data emanating from organisations’ network has hitherto received little attention. This is despite the fact that social media technologies are likely to have a tremendous effect on organisational processes of communication and collaboration, the entire process of change management (Van Osch and Coursaris 2013; Clayton 2015; Naeem 2020) in addition to providing an understanding of the nature and characteristics of those (individuals and organisations) to whom an organisation communicates to its own information output.

To be more specific, since RDI is a field that is formulated with and through by public policy as well as part of any entrepreneurial strategy, understanding the typology of individuals and organisations that follow an organisation’s LinkedIn webpage holds the potential of helping shape a more factual science, technology and innovation policy approach—to the extend, that is, followers in a social medium can be conceptualised to be a good proxy for the actual “customers” of a given organisation.

For this paper, our goal is to examine the network of our organisation, the National Documentation Centre (EKT). EKT is a Greek public organisation promoting knowledge, research, innovation in addition to seeking to enable digital transformation through these means to domestic firms and research-related individuals alike. Since 2012, it has been part of the Hellenic Statistical System responsible for the provision of the national RDI statistics. EKT is also an intermediate organisation for accessing EU funds and a data provider of scientific and cultural content, i.e. an organisation that offers services to a wide variety of customers (both public and private, entrepreneurs, policy officials and academics/researchers). It is by virtue of these evidence-focussed roles that EKT has become a backbone of the domestic RDI ecosystem. In other words, being part and parcel of the domestic science, technology and innovation policy system, we are interested in understanding who our followers are and what their characteristics are.

### Collecting data from social media

For the purposes of this paper, we are interested in understanding a network that is exclusively focussed on professionals and of which the validity of the user data can be assumed to be good (Adikari and Dutta 2020). Given this, the authors of this research paper have taken the decision of restricting this effort to Linkedin.

More specifically, in matters of collecting data from social media, and given the availability of data, a number of tools, mechanisms and techniques to retrieve and analyse LinkedIn data have been developed. Given the novelty of the field, it is important to review the available studies and methodologies already employed in the aforementioned bibliography.

LinkedIn data can be used for collecting information about one’s professional contacts such as the job title, company names or geographic locations. This can be done by applying data mining techniques for retrieving the data and performing cluster analysis for aggregating the variables of interest (Garg et al. 2016). Alternatively, such data, by constructing and validating appropriate metrics, can be utilised to explore professional skills, industry employment and talent migration (Zhu et al. 2018). Given the high volume of data, researchers have been able to even extract models of user personalities (MBTI and DISC) from Linkedin profiles (Piedboeuf et al. 2019).

### Structure of paper

The paper is structured as follows. Firstly, the research question, the paper’s potential contribution and research objectives are posed. This is followed by a demarcation of the paper’s limitations, mainly in terms of data availability and the steps taken to counter this. We then focus on the data and methods. Therein, we describe the steps to retrieve and process the data in order to obtain a clear and comprehensive dataset. The presentation of the results follows. These are presented in the following order: the network trends, sector participation and geographical location. The paper closes with the discussion and the next steps.

### Research question

It is in the above context that this paper wishes to address a related question. Specifically, by posing the question: “what is the LinkedIn network of such an organisation”, we aim to understand a range of attributes of the networked entities of an organisation—in the case of this paper, with a Greek public organisation, the EKT.

While this is an empirically based question that besets an empirically based answer that builds upon methodological (i.e. how to download, curate and process data) and statistical (identify links and networks) means and methods, it is a question with significant social media and policy analysis implications. That is, we touch upon the ever increasing exploitation of digital platforms for engaging in policy-related activities as well as how policy in matters of RDI can be best informed by way of understanding the social media network of such an organisation.

### Contribution

This paper contributes in a number of fields. For one, in this process of answering the question we make use of available methodologies of extracting, parsing, downloading, curating and analysing LinkedIn data as well as conducting analysis both at national and international level. This process presents significant interest for subsequent studies in the field—a research endeavour which will undoubtedly grow due to the increasing use of social media and the related data availability.

Given that LinkedIn is a professional-oriented networking platform (Gendronneau et al. 2019), within an organisational level, understanding the actors’ priorities, professional experience or academic background is vital for RDI potentialities. This is important as it enables an organisation to tailor its role according to its audience priorities and to develop innovative policies which fit squarely within the wider debate on knowledge and business incentives for economic and industrial growth. In view of the fact that social media data are interpreted as connected components of a community geared towards receiving intelligence (Murthy 2018; Shirky 2011), this paper is among the very few that has utilised its data in the context of RDI and as a policy tool to guide decisions within such an ecosystem.

On a parallel note, this paper provides a robust methodological framework in dealing with heterogeneous data. That is, it presents the design of methods which can be applied to extract, collect, process and analyse data, hence contributing to the wider debate on treating heterogeneous data emanating from social media (Castillo-de Mesa and Gómez-Jacinto 2020; Li et al. 2014; Aggarwal 2011). On top of that, we applied standard institutional taxonomic schemes (e.g. Frascati) to existing fields that were associated with non-standardised entries.

Further, this paper contributes in the wider bibliography concerning social media as informational outlets contributing to policy analysis as well as digital platforms contributing to institutional change.

On this, by way of understanding the nature and characteristics of entities (individuals and organisations) to which an organisation communicates its own information output we contribute to the wider discussion of social media technologies having an impact on a range of institution’s organisational parameters and processes. More specifically, since RDI is a field that is formulated with and through by public policy as well as part of any entrepreneurial strategy, understanding the typology of individuals and organisations that follow an organisation’s LinkedIn webpage holds the potential of helping shape a more factual science, technology and innovation policy approach. Moreover, in addition to standard RDI indicators, data extracted from social media can be used as alternative metrics (Wouters et al. 2019; Wiechetek and Pastuszak 2022). Indeed, this practice has been on the rise (e.g. Baruffaldi et al. 2017; Ernst and Brem 2017; Zheng and Zheng 2014; Sugimoto and Larivière 2016; Wang et al. 2016).

Zooming out of RDI policy, establishing a more factual analysis of social media data holds the potential of providing a more ‘grounded’ and evidence-based approach as a way out and counter-weight of (dis- and mis-) information activities.

### Research objective

The objective is to examine the network of our organisation, by way of analysing data from Linkedin. That is, to explore our connections’ profiles as indexed in the platform of LinkedIn. Herein, by the term “profile” it should be taken to mean a list of specific attributes that are linked to each individual that “follows” EKT. Similarly, the term “follow” is perceived as the conscious connection between EKT and any individual/organisation—a connection that is established by the latter side.

Such exploratory attributes are, to begin with, the basic information of each user, such as their first name, last name and physical address. This information is submitted by each user to the LinkedIn platform as a means to make known exactly this set of attributes. In addition, Linkedin’s employment agenda, with the provision of the name of the connection’s employer, along with the job’s title. Also, the platform provides the date of the established connection—i.e. the date that the user chose to connect with—or, in our case as explained before, to follow ΕΚΤ.

### Limitations

LinkedIn, as a company, abides by the rules set by industry-specific needs. As such, data availability is dependent upon company policy issues. This is a standard, yet an important primary limitation. Additional limitations concern the homogeneity of data, the design of the application’s user interface, blank or over filled columns, abbreviations, misspelled, etc. (Garg et al. 2016).

As aforementioned, the platform includes employers posting jobs and job seekers sharing their education and employment history. Such data would be helpful to compute meaningful migration estimates for different education levels and job categories. However, during the course of this analysis it became evident that we could not have access to these data. The data available through LinkedIn API allows filtering by age, gender, education level and sectors. However, the platform does not allow filtering based on country of origin. Although it is possible to filter by university attended it does not seem to be a reliable proxy regarding the country of origin. In other words, a far too great number of assumptions would have to be made. This is unfavourable if, for example, one takes into consideration the Erasmus Programme which funds student mobility among universities. This limitation is also corroborated by Gendronneau et al. (2019).

Another limitation pertains to the network of LinkedIn connections. People who join LinkedIn are principally interested in the business opportunities that it provides as opposed to arbitrary socialising and will necessarily be providing sensitive details about business relationships, job histories, conducted projects and more. In turn, a valid hypothesis would be that these details would have been cautiously edited by the user in the first place.

On behalf of LinkedIn, certain limitations concerning data availability have been intentional. For example, while one can generally access all of the details about LinkedIn connections, educational histories, and previous work positions, one cannot determine whether two arbitrary people are “mutually connected”. The absence of such an API method is intentional. The API does not lend itself to being modelled as a social graph like Facebook or Twitter. Therefore, this requires to set different types of questions about the available data (Russell and Klassen 2019).

### Data and methods

For an individual user of LinkedIn with a profile it is rather simple to retrieve his/her relevant connections. One can simply navigate to the settings and privacy section of their profile and click on the “request archive” button (Tavish Gobindram 2020). However, EKT being an organisation is represented by a LinkedIn page which gains “connections” when followed. Unfortunately, Linkedin’s policy on data retrieval from such pages is strict and thus relevant data cannot be extracted or downloaded in a manner as compared to individuals. Herein, a problem was encountered. To address this, we attempted to identify the standard manner in which the related bibliography approaches the issue at the organisation level. However, the search results were insufficient. In fact, no analogous study could be identified in the current literature.

After thorough research it became evident that accessing the content API of LinkedIn^1^ was a prerequisite in order to retrieve the variables of interest. As such, the authors created an application^2^ (15/03/2021) that requested permission from LinkedIn for the the full-profile fields.^3^However, no answer was provided by LinkedIn. As an alternative, within the Python (3.9) environment, the authors implemented an algorithm that could scrape all the variables of interest for each individual. However, taking into account LinkedIn’s user agreement,^4^ intricate legal issues and considerations (Sobel 2020) as well as limitations on the volume of data that can be scraped (Russell and Klassen 2019), the authors decided that was optimal—timewise—to proceed with a different approach (see following sections).

The data collection and the overall analysis were conducted between March and May 2021. Due to the fact that the period in which data were harvested was early 2021, the data concerning the number of followers for 2021 are not included in the analysis.

#### Data Retrieval—Part 1

As regard profile description, although LinkedIn suggests specific attributes for each provided field,^5^it is entirely up to each individual which attributes to submit within each field. Because of this characteristic, for the purposes of this paper, it was assumed that a lot of ‘noise’ would be found therein. In view of this, Table 1 elucidates this issue in accordance with the empirical findings of this study.

**Table 1:** LinkedIn’s fields and their interpretation according to the objects of study

Profiles (LID)	The LinkedIn ID associated with each user
Names	The user name as it appears in the LinkedIn account
Headlines	The LinkedIn section where individuals introduce themselves. Usually contains information about the current position and current experience
Current Position	The “current position” refers to employment positions. For example ‘Software Developer’, ’Professor’, ‘PhD candidate’, etc
Current Experience	The “current experience” refers to an individual's spatial position. It usually names organisations such as firms, universities, research centres, etc
Physical Address	The “current country” that individual is geographically located in
Followers	The individuals/organisations that follow EKT’s LinkedIn page
Followed at (month)	The date (month,year) that an individual established a connection with (followed) the organisation

In order to retrieve data relevant with our organisation, super admin credentials were needed. As such, within a “super admin view” we searched for information related to our page followers.

A relevant list was displayed (EKT > Analytics > Followers > See all followers) containing followers’ names and headlines. The “Headline”’ attribute includes the current position and current experience (where existing) of the individuals. In order to obtain a comprehensive view of the entire catalogue of followers we scrolled down the list.

Secondly, in an effort to obtain a parsable html file containing relevant characteristics with our object of study—First name, Last name, Linkedin ID, Headline, Followed at (month)—the command “inspect element” was utilised.

Using a Python script that could parse the html file, we managed to construct a comma separated value (.csv) file including all the aforementioned variables. The total number of users’ profiles collected amounted to 5.273.

#### Data processing

**Fig. 1:**
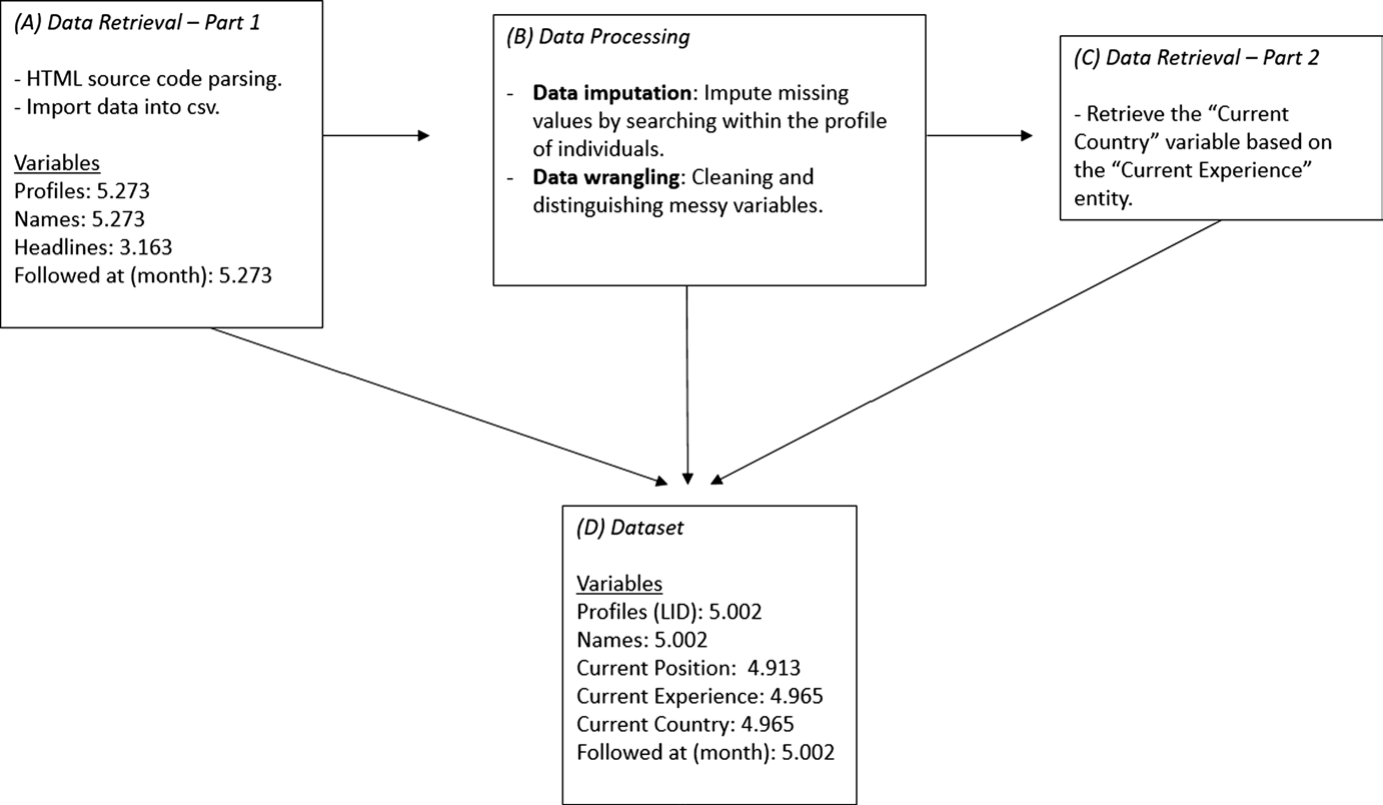
The sequential steps involved in the data collection process

Exploring the data, no missing values were found with regard to followers’ First name, Last name, LinkedIn ID as well as month of the established connection—“Followed at (month)” variable (see Fig. 1). This was not the case regarding the “Headline” variable. In fact, there was an approximate 40% of the rows (*N* = 2.110 individuals) for which the headline manipulation failed to give us a job title and/or employer. This was because the aforementioned users did not have an appropriate headline for their profile filled in.

#### Data imputation

As aforementioned, the “Headline” variable usually contains information about the “current position” and the “current experience” (job title, organisation etc.) of the individual i.e. “Data Engineer at Microsoft”. However, although (as any field in LinkedIn) it is optional to be filled in, in order to confirm the (non)-existence of the “current position” and “current experience” further examination took place.

In fact, the authors undertook the manual process of probing into LinkedIn’s individuals profile (utilising the LinkedIn id of each individual) and searching for information in LinkedIns’ fields “Experience” and “Educations”. This was conducted for the aforementioned 40% of the individuals with no filled in “Headline”—all “Headlines” have been successfully retrieved. Thus, additional information with respect to followers’ position and job title were collected.

#### Data wrangling

With the aim of constructing a more concise database, we decided to break down the “Headline” variable into two distinct features; “current position” and “current experience”.

A good starting point in our attempt to clearly separate the “Headline” variable into the two distinct variables was the fact that for approximately 87% of the entries the text referring to the “Headline” feature, contained the word “at” (the syntax was current position “at” current organisation). Therefore, using basic excel functions the two variables were successfully separated. This resulted in 4.587 entries for each variable. For the remaining entries (*N* = 686)—the syntax was “*current position “,”* *current organisation*” or “*current position “”* *current organisation*”—the “current position” as well as the “current organisation” were manually distinguished.

At this point in the finalisation of the data, there was a persistent number of rows/users for which it was impossible to recuperate a job title or an employer name. Some of these users had a number of ASCII characters that were neither Greek nor English letters (*N* = 179 profiles), while others had Linkedin Profiles which were not meticulously made; they did not contain information neither about the “current position” nor the “current experience” (*N* = 129 profiles). These rows (*Ν* = 308) were deleted, resulting in 4.965 entries regarding the “current experience” and in 4.913 with respect to the “current position” variable (see Graph 1). The difference between the number of entries with respect to the “current position” and “current experience” variable is attributed to the fact that regarding the former variable there were additional missing values (*N* = 52 profiles).

#### Data retrieval—Part 2

With regard to the collection of the variable that pertains to the physical address of the individuals, a separate approach was followed. By utilising the “current experience” of the users (see Table 1), the authors were able to determine the corresponding location—i.e. the “current country”—in the following manner. Assuming that the current experience of a specific user takes place at the “National and Kapodistrian University of Athens”, the corresponding, current country that the individual is located in is Greece.

Following the same reasoning, the “current country” as concerns the majority of the cases could be smoothly extracted (78.3%). However, with respect to the remaining cases (21.7%), the corresponding country could not be easily recognised; cases in which solely the company name of the individuals was present i.e. ING, Amazon, Google, etc. For such cases, additional examination took place within the LinkedIn profile of each individual in order to retrieve the corresponding country. Complementary, desktop analysis including search engines in tandem with other internet sources (mainly webpages) were utilised.

For each entry of the countries collected, the alpha—3 letter code was used.^6^ Therefore, 4.965 such entries were collected.

#### Dataset

The final dataset consists of 5.002 LinkedIn profiles. However, for the purposes of this study only specific variables were considered. That is, the overall analysis was performed having as a reference point the entries of the “current experience” variable (*N* = 4.965). The “current position” profile feature of the individuals was not utilised for further analysis.

## Analysis and Results

### Part 1 Network trends

**Fig. 2:**
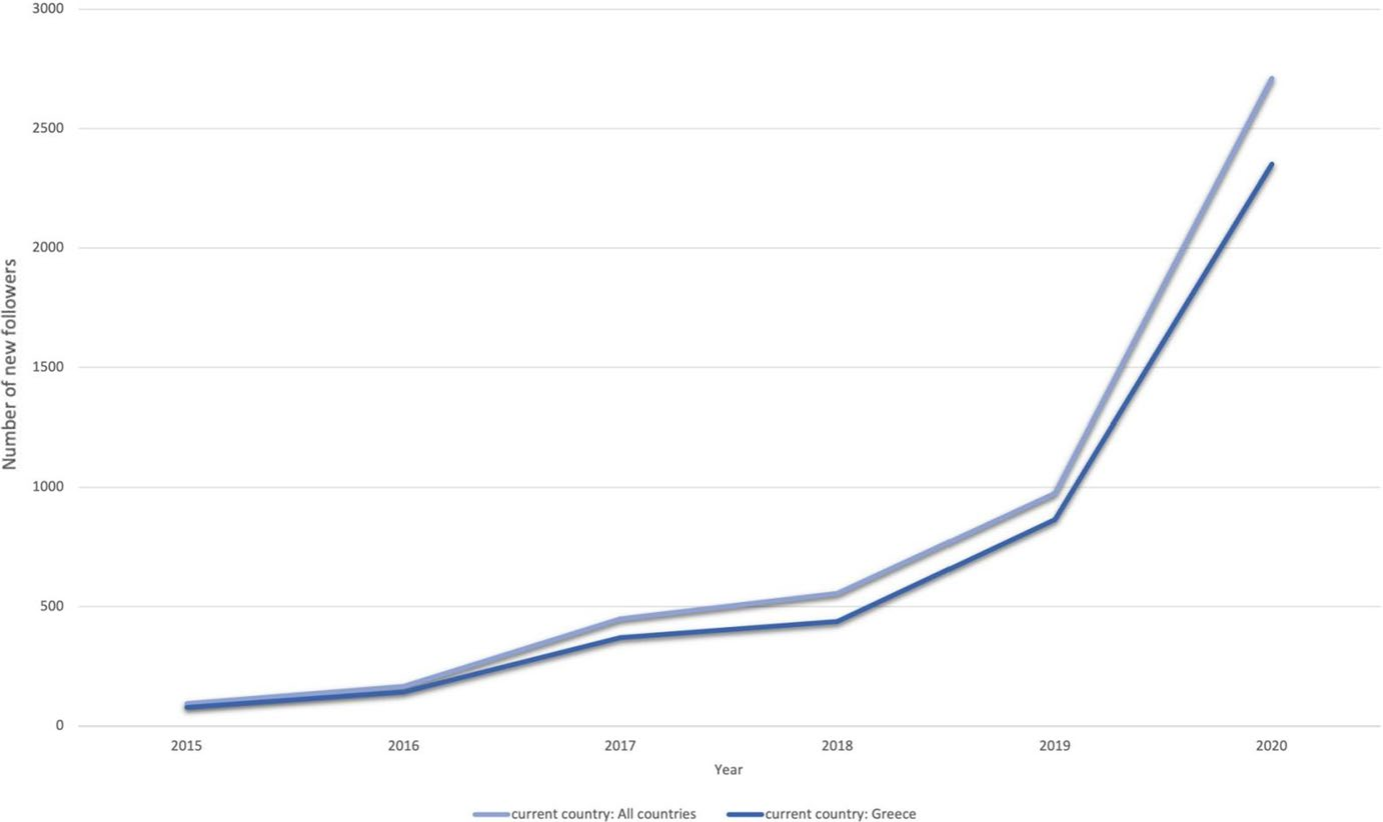
The distribution of the “current country” of individuals (see Table 1) that follow EKT’s LinkedIn page (followers) throughout the period 2015–20

The following figure (Fig. 2) presents the number of individuals following EKT’s LinkedIn page for the period 2015–2020. The two timelines indicate followers that are located within Greece, whereas the second records the increase on the global scale. Cumulatively, the portion of the individuals that follow EKT’s LinkedIn page and have Greece as their “current country» amounts to 85.6% of the total (*N* = 4.249 individuals), whereas the remaining 14.4% (*N* = 716 individuals) are located in other countries. Over time, the number of new follows was increasing. What started with a few tens of followers in 2015 it has grown to more than 2.500 in 2020. In fact, there is an apparent growth in the number of followers between 2019 and 2020, which amounts to *Ν* = 2.713 new followers. This number corresponds to the 54.7% of the total number of followers during the six-year period of 2015–20. Such a rapid increase can be attributed to the fact that during the start of pandemic, and, by the time that the World Health Organization (WHO) convened a “Research and Innovation Forum” on COVID-19 (WHO 2020), EKT, following the principles of open science, constructed a portal that aimed at disseminating all available COVID-19-related scientific information in any interested communities (Sachini et al. 2021). This may potentially account for the attracted interest. While the majority of followers are located in Greece, the number of followers from foreign countries has been increasing also.

### Part 2 Geographical location

In order to schematically point out the evolution of the international network of EKT formed by the LinkedIn followers, a world map containing the “current countries” in an annual basis manner is presented. The map was constructed taking into consideration the “current country” (see Table 1) that was associated with the per year number of followers.

**Fig. 3:**
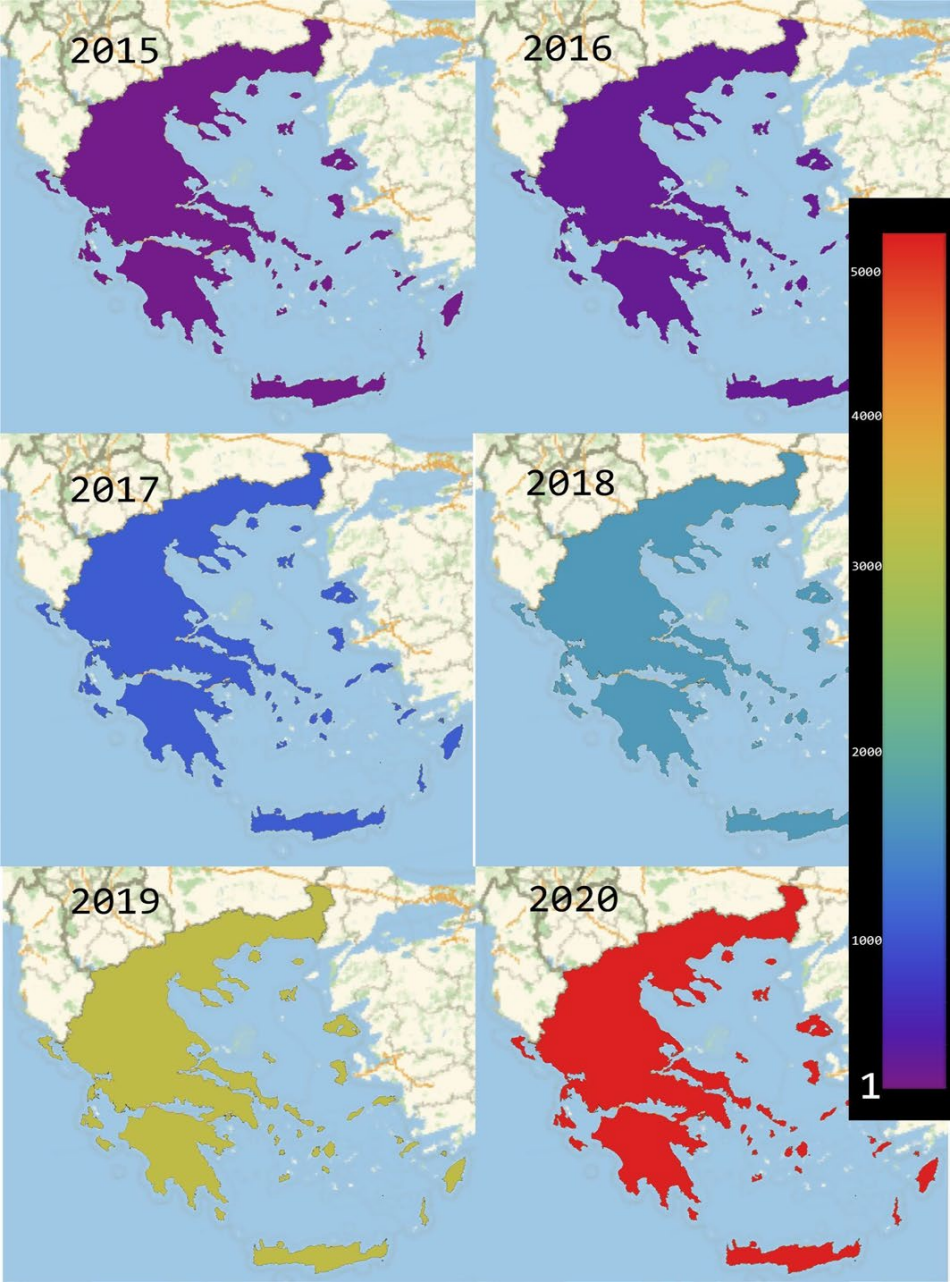
The annual evolution of EKT’s followers over the period of 2015–20, having as “current country”: Greece

Figure 3 presents evidence of the annual evolution of EKT’s LinkedIn followers which have Greece as their “current country”. It is evident that between 2015 and 2020, ΕΚΤ’s LinkedIn page has been gaining *publicity* and has attracted more than 4.000 followers. With an increasing array of scientific and policy output (e.g. statistics, reports, publications, infographics) in scientific and policy matters that refer to RDI as well as developmental and economic matters, EKT has been actively seeking to disseminate this material (and) through LinkedIn. In return, the increase in the number of the LinkedIn’s followers reflects both the interest into this kind of informational output as well as the growing recognition by the domestic audience as an established RDI organisation.

**Fig. 4:**
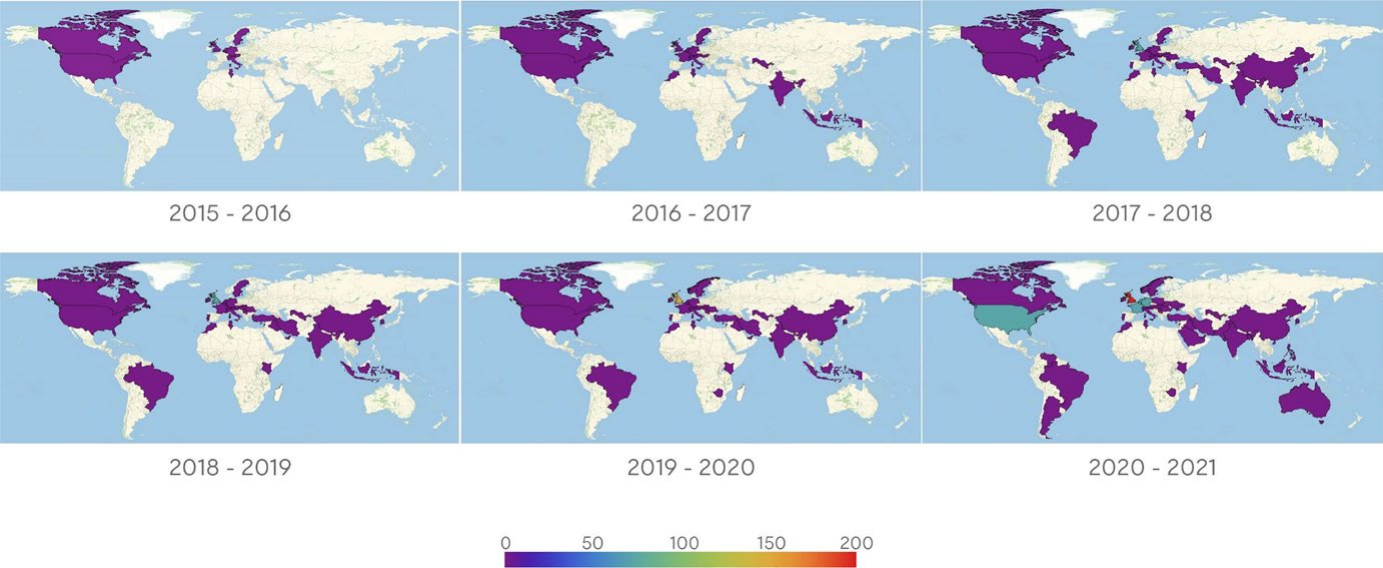
The annual evolution of EKT’s followers over the period of 2015–20, having as “current country”: not Greece

Figure 4 presents evidence of the annual evolution of EKT’s LinkedIn followers which have as their “current country” countries other than Greece. This level of analysis allows the reader to explore the footprint of the followers at the country level; the colour in each country corresponds to the cumulative number of followers for the specific year as shown in each legend. Observing the worldwide maps, 68 countries in total followed EKT over the time period of 2015–20. As a general trend, one can visualise that as time goes on, more countries are added to the international network map (peaking in 2020). Also, for the following countries: United Kingdom, the United States, France, Germany, Belgium, the Netherlands and Cyprus the change in the colouring denotes a significant increase in the number of followers. It is evident that those countries constitute the “current countries” with the greatest number of followers. In fact, more than 150 followers have been associated with the United Kingdom as their “current country” throughout the six-year period of 2015–20. The remaining aforementioned countries attract 60–90 followers.

### Part 3. Sector participation

For the purposes of understanding the national as well as international actors that participate in our organisation’s network, the institutional sector of the population is presented in the following part. Specifically, given that the institutional classification of those following EKT was sought, we extracted the relevant institution categories from the “current experience” feature. The information contained therein, was, then, distributed manually^7^ to the following institutional sectors: HES, GOV, BES, PNP following OECD’s accepted Frascati taxonomy (OECD 2015). It is to be reminded that:

–The Higher Education Sector (HES) includes Universities, Technological Educational Institutes (TEI) and Other Higher Educational Institutions.
–The Government (GOV) sector pertains to Public Hospitals, Research Centres supervised by the General Secretariat for Research and Technology (RC-GSRT), Other Public Research Institutions, Other Public Institutions, etc.
–The Business Sector (BES) consists of Enterprises as well as Private Health Institutions.
–The Private Non-Profit Sector (PNP) is self-explanatory.

It is to be noted that a numerically insignificant minority (*N* = 16 individuals, 0.3%) had declared as having multiple “current experience”. For example, “Software Developer at Google/PhD at University of Oxford”. For these cases, we considered only the firstly mentioned experience—“Google” for the case of the example. Also, 7 cases were not classified, since their current experience had been declared as “Unemployed”. As such, 4.958 (*N* = 4.965–7) were categorised into the aforementioned sectors. The above classification enabled the analysis and allowed the exploration of each institutional sector.

**Fig. 5:**
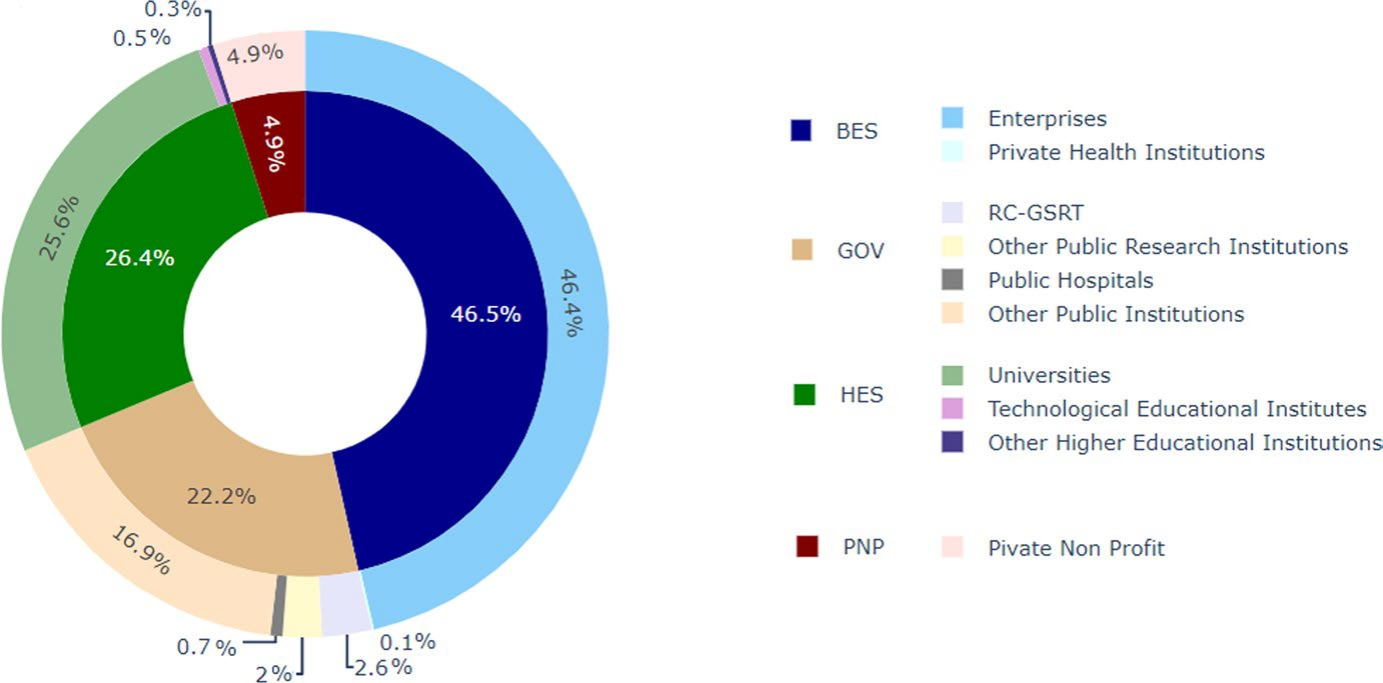
The distribution among institutional sectors and sub-sectors across the target population. Such a population consists of the total number of followers (*N* = 4.965) both having their “current experience” in Greece and in other countries

Figure 5 presents the sector classification of all individuals following EKT’s LinkedIn page. The inner circle presents the distribution among the 4 institutional sectors, whereas the outer circle presents the sub-sectors falling under each institutional sector*.*

The greatest portion (46.5%) of the individuals is categorised into the Business sector (BES). Within BES, most (46.4%) belong to the sub-sector of enterprises (Amazon, Microsoft, PwC, etc.), whereas 0.1% are categorised as “Private Health Institutions” (Mitera Hospital, London Women’s Clinic, etc.).

The 26.4% of the individuals’ institutions was classified into the Higher Education sector (HES) with the vast majority of the sector coming from Universities (25.6%), 0.5% from Technological Educational Institutes and 0.3% from Other Higher Educational Institutions.

Concerning the Government sector (GOV), 22.2% of all individuals fall within this sector. Of those, 16.9% belong to the sub-sector “Other Public Institutions”. Examples of such institutions include Ministries, public schools, etc.

2.6% report that they belong to the Research Centers of the General Secretariat for Research and Technology, whereas 2% classify themselves to the Other Public Research Institutions^8^ and 0.7% to Public Hospitals.

The remaining 4.9% concerned institutions belonging to the Private non-Profit (PNP) sector.

Given that very few, if any, have analysed the institutional classes of a given set of followers of an organisation before, it is difficult to comment on the presented institutional distribution in terms of past bibliography and findings. However, given the nature of EKT—the national statistical agency on RDI, an intermediate organisation for accessing EU funds, a data provider of scientific and cultural content, i.e. an organisation that offers services to a wide variety of customers (both public and private, entrepreneurs, policy officials and academics/researchers), this mixed institutional distribution recorded in Fig. 5 can be potentially accounted for.

**Fig. 6:**
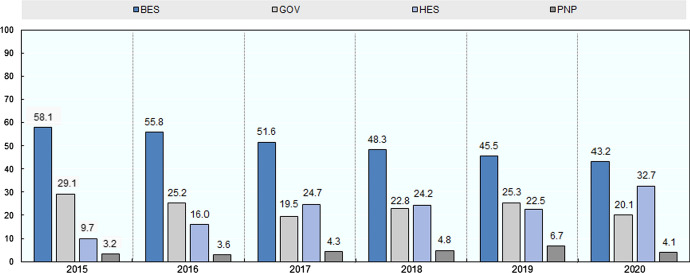
The distribution of the institutional sectors (BES, GOV, HES, PNP) across the time period 2015–20

In addition to understanding the overall distribution of the institutional classes of the individuals that have followed EKT’s page, analysis of the distribution for the period 2015–20 on a yearly basis is presented in Fig. 6.

A number of findings arise. Firstly, for the given time period, the superiority of the BES sector is evident. However, given its steady decrease over time (from 58.1% in 2015 to 43.2% in 2020) it would not be surprising for BES, down the road, to fall into second place after HES. Indeed, individuals from HES have almost quadrupled, stretching from 9.7 to 32.7%, within the same time frame.

With regard to the GOV sector, the relevant portion within this six year period ranges from 20 to 29% of the total.

Last are individuals from PNP organisations (3–7%).

It is difficult to point to specific conditions that can explain the increase and/or decrease of the number of followers over time. This is made further difficult given the multi-role character of EKT. A potential indication of the increase of HES followers can be ‘tied’ to the availability of data for conducting secondary RDI analysis on the national and regional level as well the availability of scientific information concerning COVID-19 through a dedicated portal.

**Fig. 7:**
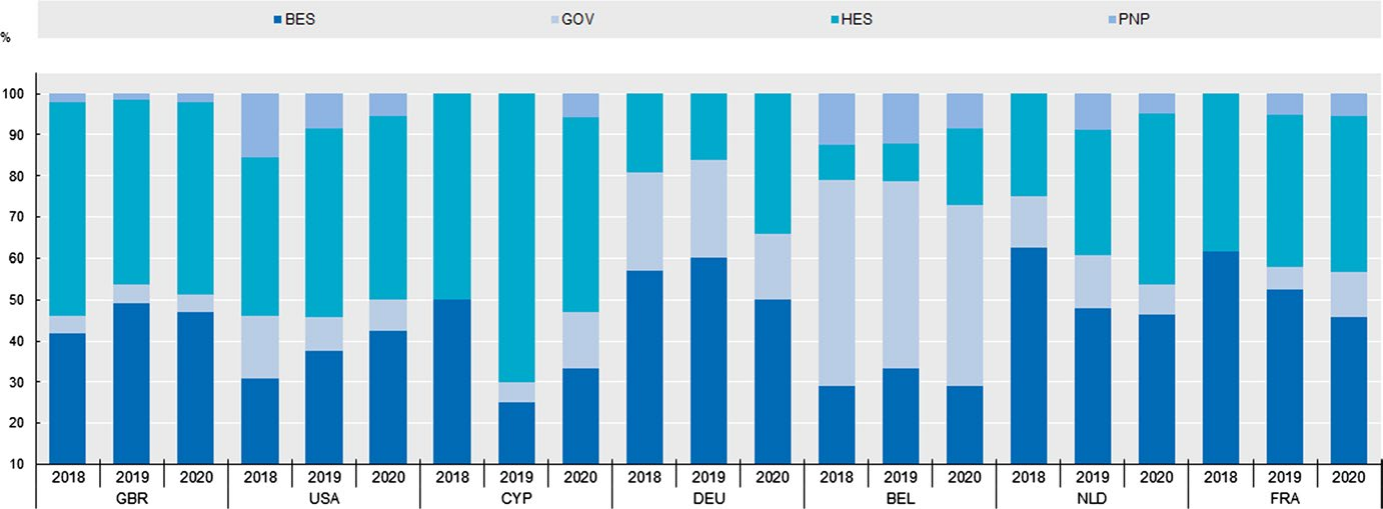
Frequency distribution of the institutional sectors (BES, GOV, HES, PNP) with respect to the countries with more than 50 followers in total (top 7 countries). Time period: 2018–20

Concerning the institutional classification of EKT’s international followers, Fig. 7 presents their frequency distribution. A threshold was set for visualising only those countries in which more than 50 followers are found. This is the case for 7 countries and for the period between 2018 and 2020.

With the exception of the USA, all the remaining 6 are European countries (UK, Germany, Belgium, the Netherlands, France, Cyprus). Countries in Asia, Africa and Latin America do not form part of this group.

The BES and HES sectors constitute the majority of followers. Despite yearly fluctuations, this is the case for most countries. Exception to this is the case of Belgium. Therein, the GOV sector dominates the distribution (50% in 2018, 45.5% in 2019, 43.8% in 2020). Looking at the data, this is due to the fact that most of EKT’s followers in Belgium report as their “current experience” European organisations (European Commission, European Research Council, European Students'​ Union (ESU), Permanent Representation of Greece to the EU etc.). This is understandable given EKT’s role as a domestic intermediate organisation responsible for, among other things, enabling the participation of Greek institutions to European RDI projects. As such, EKT has established links to European organisations—something that is mirrored herein.

## Discussion

There are a number of topics that deserve discussion. For one, the use of cutting edge techniques to curate and analyse data. While reminded of the fact that LinkedIn is a private firm, the ability of users to upload their information with complete disregard to any taxonomic scheme in relation to fields of education, employment, etc. incapacitates most attempts to execute secondary analysis on the typology of followers. That is, analysis which is consistent and referential to past analyses must beforehand be thoroughly classified according to standard classifications. To accomplish this, one avenue is to exploit advanced data science techniques (text classification, clustering, etc.) that could be of value. However, such approaches come with a cost in accuracy and presuppose a standard manner upon which the entire, heterogeneous data are modelled; one must go through the whole text, analyse it and then a set of pre-defined labels must be assigned (in terms of text classification). In the case of this paper and in mind of the speed-accuracy tradeoffs, due to the manageable amount of the data, the authors undertook a significantly manual approach as regard data homogenization following standard taxonomic schemes (e.g. Frascati).

Conversely, this case provided data cleansing experience. Given the increasing availability of alternative metrics for measuring RDI policy, mainly referring to social media metrics, in addition to data linking as the new global trend understanding the typology of the followers brought understanding in relation to data cleansing issues. Appreciating the different characteristics of the LinkedIn data will provide significant insights in relation to the homogenising different datasets through, e.g. the transliteration to common taxonomic schemes.

In matters of enhancing policy analysis and STI policy, in addition to standard RDI indicators, social media have been used as alternative metrics. Their influence on RDI practices is on the rise (e.g. Baruffaldi et al. 2017; Ernst and Brem 2017; Zheng and Zheng 2014). Within scholarly communication, social media have been also utilised as indicators of broader impact of scientific publications and of other research products (Sugimoto and Larivière 2016; Wang et al. 2016). Similarly, it can inform on the nature and typology of the network of a specific organisation. In this case, it was shown that the followers of EKT form part of a mixed group consisting of academics and researchers, entrepreneurs and governmental officials. Potentially, a time-dependent analysis of the number and rate of new, linked individuals and organisations can be exploited as a new, additional, metric for recording the attractiveness and perceived authority of any RDI-related organisation.

In the case of EKT, such as this mixed typology of individuals and organisations is most probable attributed to the nature and complexity of its core missions—that is, being a statistical agency, an intermediate organisation for accessing EU funds and a data provider of scientific and cultural content. Most of EKT’s LinkedIn network is recorded within Greece. This finding should be appreciated and relevant steps should be taken for increasing the organisation’s global social media footprint. Obviously, this should be done in sync with the organisation’s complementary social media means, such as Twitter. A more active posting rate of english-language material and a more active approach towards the Greek science diaspora are two potential avenues. Obviously, this place-based parameter is something to be incorporated to the aforementioned alternative metric discussion.

Wider understanding of these data enables a more hands-on approach on tailoring ones’s own strategy and approach role according to its audience priorities and to develop innovative policies which fit squarely within the wider debate on knowledge and business incentives for economic and industrial growth. Building on Murthy (2018) and Shirky (2011), social media data being connected components of a community, findings herein explored and identified what is the form and shape of the domestic RDI ecosystem and, as such, offered a first clue of actual evidence to guide policy decisions within such an ecosystem. Zooming out of RDI policy, establishing a more factual analysis of social media data holds the potential of providing a more ‘grounded’ and evidence-based approach as a way out and counter-weight of (dis- and mis-)information activities.

### Further research

In addition to LinkedIn, analysis of other social media is a future avenue. Different datasets as well as different data cleaning methods will increase our understanding and capacity to handle data coming from various sources. Parallel analysis of these sources will reveal complementarities as well as incompatibilities in relation to our organisation’s place in the respective networks. Mapping the LinkedIn network of other pillars of the domestic RDI system (e.g. funding agencies) will provide a picture of the domestic RDI system as taken from a different “angle”.

Obviously, work that builds on this paper and examines similar agencies on the global scale will help validate/refute our findings.

## Data Availability

The sensitive nature of these data means that they are only available internally. For external researchers, ethical approval may be obtained via formal request and application to the National Documentation Centre (EKT) for a specific research project. Interested parties are advised to contact the corresponding author (nkarampekios@ekt.gr) to discuss the application.

## Footnotes

1 https://docs.microsoft.com/en-us/linkedin/learning/getting-started/request-access.

2 https://learning.linkedin.com/partners/partner-application.

3 https://docs.microsoft.com/en-us/linkedin/shared/references/v2/profile/full-profile?context=linkedin/consumer/context.

4 https://www.linkedin.com/help/linkedin/answer/56347/prohibited-software-and-extensions?lang=en.

5 https://docs.microsoft.com/en-us/linkedin/shared/references/fields.

6 https://www.iban.com/country-codes.

7 For example, the “National Technical University of Athens” is categorised into the HES sector under the subsection “Universities”, the “Ministry of Digital Governance” is categorised into the GOV sector under the subsection “Other Public Institutions”, “Google” is categorised into BES under the subsection “Enterprises” and “Stavros Niarchos Foundation Cultural Center (SNFCC)” is categorised into PNP.

8 It is to be noted that the share of individuals employed in EKT—classified as GOV subsection “Other Public Research institutions”—amounts to 45.7% of this subsection (*N* = 47 individuals).
